# An Overview of Equine Influenza in South America

**DOI:** 10.3390/v13050888

**Published:** 2021-05-12

**Authors:** Cecilia Olguin-Perglione, María Edith Barrandeguy

**Affiliations:** 1 Instituto de Virología CICVyA, Instituto Nacional de Tecnología Agropecuaria (INTA), Hurlingham B1686, Argentina; barrandeguy.maria@inta.gob.ar; 2 Escuela de Veterinaria, Facultad de Ciencias Agrarias y Veterinarias, Universidad del Salvador, Pilar B1630AHU, Argentina

**Keywords:** equine influenza, South America, H3N8, H7N7

## Abstract

Equine influenza virus (EIV) is one of the most important respiratory pathogens of horses as outbreaks of the disease lead to significant economic losses worldwide. In this review, we summarize the information available on equine influenza (EI) in South America. In the region, the major events of EI occurred almost in the same period in the different countries, and the EIV isolated showed high genetic identity at the hemagglutinin gene level. It is highly likely that the continuous movement of horses, some of them subclinically infected, among South American countries, facilitated the spread of the virus. Although EI vaccination is mandatory for mobile or congregates equine populations in the region, EI outbreaks continuously threaten the equine industry. Vaccine breakdown could be related to the fact that many of the commercial vaccines available in the region contain out-of-date EIV strains, and some of them even lack reliable information about immunogenicity and efficacy. This review highlights the importance of disease surveillance and reinforces the need to harmonize quarantine and biosecurity protocols, and encourage vaccine manufacturer companies to carry out quality control procedures and update the EIV strains in their products.

## 1. Introduction

Equine influenza (EI) is considered, in economic terms, one of the most important respiratory diseases of horses and other equids. This is due to the highly contagious nature of the causative agent, the equine influenza virus (EIV) [1,2,3]. EIV is endemic worldwide except for Iceland and New Zealand, where it has never been reported, and Australia, which is currently free again, after the first incursion of EI in 2007 [2,4,5]. Outbreaks of EI are associated with the commingling of horses for competitions and other equestrian events and with the international movement of horses [4,6,7].

Influenza A viruses are subtyped according to their surface glycoproteins, hemagglutinin (HA) and neuraminidase (NA) [8]. Two subtypes of influenza A virus, the H7N7 and the H3N8 have been associated with respiratory disease in horses [9,10]. Although the H7N7 EIV was first isolated in 1956 in an outbreak of the respiratory disease in horses in Czechoslovakia and circulated in horse populations for approximately 25 years, it is currently considered extinct [9,11]. The H3N8 EIV subtype was first detected in 1963 during a major epizootic of respiratory disease in horses in Miami, Florida, United States (USA), and ever since, this subtype has circulated continuously among horse populations, being responsible for EI outbreaks worldwide [2,12,13,14]. According to phylogenetic analysis of the hemagglutinin (HA) gene, the H3N8 EIV evolved as a single lineage for at least two decades and diverged during the mid-1980s into two different lineages American and European [12,15,16]. Subsequently, the American lineage diverged into South American, Kentucky and Florida sublineages [17]. In the early 2000s, the Florida sublineage evolved into Florida clade 1 and Florida clade 2 and has been the predominant circulating sub-lineage since then [12]. Florida clade 1 virus has been isolated in North America since 2003 and has been associated with EI outbreaks in South Africa, Japan, Australia, Europe, the United Arab Emirates, South America and more recently in Africa [12,18,19,20,21,22,23,24,25,26,27,28,29]. Florida clade 2 EIV strains are the predominant EIV strains circulating in European and Asian countries, and although it has been isolated in horses imported into the United States, it has never been detected in South America [19,20,30,31,32,33,34,35,36].

Vaccination is widely used to prevent or limit the disease’s spread, strength, and negative impact [2,37]. However, like other influenza A viruses, EIV undergoes antigenic drift by accumulating amino acid substitutions in the HA protein, which has led to vaccine breakdown [23,38,39,40]. Consequently, the composition of EIV vaccines needs to be reviewed and updated periodically. The World Organization for Animal Health Expert Surveillance Panel on Equine Influenza Vaccine Composition (OIE-ESP) makes annual vaccine strain recommendations based on the genetic, antigenic and epidemiological information of EI field outbreaks occurring worldwide. Since 2010, the OIE-ESP recommendations have included both Florida clade 1 and 2 sublineage strains in EI vaccines [6,41].

Considering the importance of understanding the epidemiology of EIV and the knowledge of the strains that circulate in different regions worldwide, in this review, we summarize the scientific and anecdotal information available on equine influenza in South America.

## 2. Main Outbreaks of Respiratory Disease due to EIV Infection in Horses in South America

The EIV of the H7N7 and the H3N8 subtypes were responsible for numerous episodes of febrile respiratory disease in horses in South American countries, such as Chile, Brazil, Uruguay, Argentina, Colombia and Ecuador, where the equine industry plays a critical role in generating jobs and income. EI is considered endemic in many countries in the region, while in others, there are no cases reported. The fact that some South American countries have never communicated the occurrence of EI could be due to the absence of the disease itself or the lack of surveillance and specialized diagnostic laboratories. It is also important to highlight that the horse industry with valuable competitive horses is limited to some countries, as in Brazil and Argentina, the most important is the number of animals. Brazil is home to approximately 5.7 and Argentina to 2.7 million horses and other equids. Though many of these horses belong to native breeds, Thoroughbreds and other sport horses are of great importance in these countries (https://ker.com/equinews/horse-industry-brazil/, accessed on 27 April 2021; https://www.magyp.gob.ar/sitio/areas/equinos/informacion_estadistica/, accessed on 27 April 2021). The thoroughbred industry in South America shares common objectives to develop and protect the industry through the “Organización Sudamericana de Fomento del Sangre Pura de Carrera” (OSAF). The members of this organization are institutions related to Thoroughbred horses, from Argentina, Uruguay, Brazil, Perú, México, Venezuela, Chile, Colombia, Paraguay, Ecuador and Panamá (https://www.osafweb.com.ar/en/members/, accessed on 27 April 2021).

**Figure 1:**
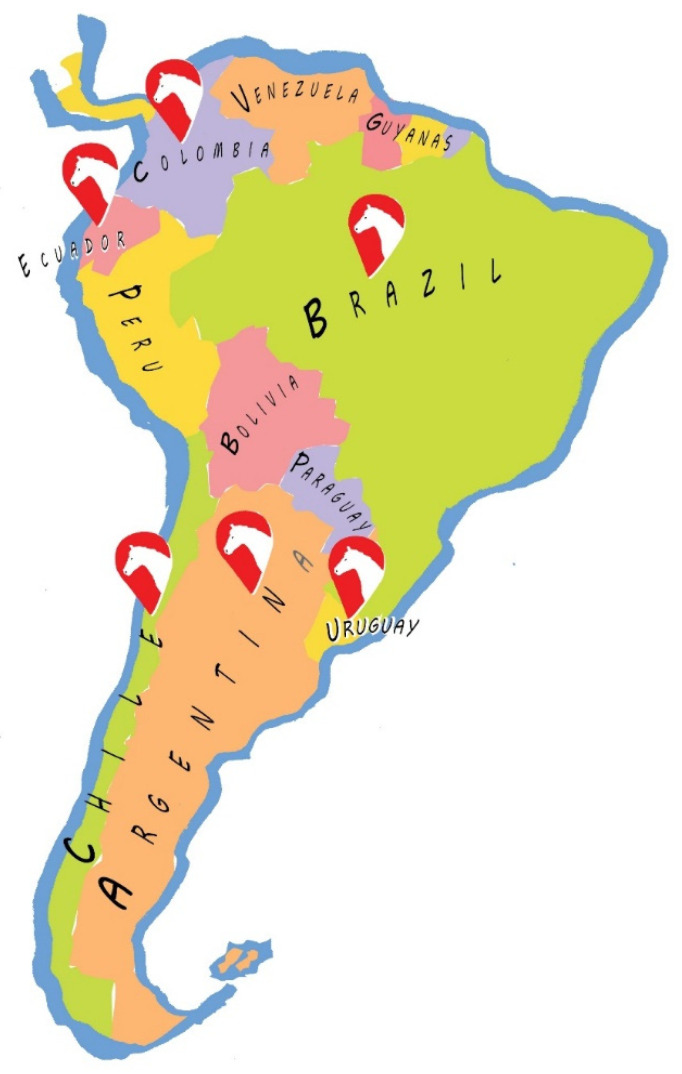
Geographical localization of the countries where EIV was reported in South America.

The geographical distribution of the countries in South America where EI outbreaks were described and reported is summarized in Figure 1 and Table 1.

### 2.1. Chile

The first registered occurrence of EI disease in South America was reported in Chile in June 1963, characterized by an acute presentation and rapid dissemination. The outbreak was due to an EIV H3N8 subtype and occurred almost simultaneously with the first described case of the EIV H3N8 in Miami, Florida, USA [14,42,47,55]. A further event of EI took place in the summer of 1977, caused by an EIV H7N7 subtype and characterized for being clinically more severe than the previous one [46]. The EIV H3N8 subtype re-emerged during summer 1985–1986, spreading from Central to Southern Chile [47]. Ever since all the EI outbreaks have been due to the H3N8 EIV, the outbreaks that occurred in 1992 and 2006 involved both vaccinated and unvaccinated horses [53]. In January 2012, EIV was again confirmed and reported to the World Organization for Animal Health (OIE) (https://wahis.oie.int/#/home, accessed on 4 March 2021) [24]. In January 2018, several horses were compromised in the last known EI occurrence in Chile. The epidemic started in Central Chile and rapidly spread across the country, virus transmission being favored by the Chilean rodeo qualifiers season, which implied an increase of horse movement, and also by the unvaccinated status of the horse population. In this country, mandatory EI vaccination was imposed recently, in 2020 [54,56].

### 2.2. Brazil

A brief mention of the first evidence of the circulation of EIV H3N8 in Brazil in 1963 is made by Favaro et al., 2018 [43]. These authors also describe a severe respiratory disease event due to EIV H7N7 subtype infection that occurred in 1976 [43]. Some other EI outbreaks due to EIV H3N8 subtype infection were described as occurring in 1969, 1986, 1988, 2008 and 2010 [25,49,57].

An extensive onset of acute respiratory disease in horses was reported in 2012 [25]. According to this report, the first clinical cases were observed in February, following an International Creole Rodeo held in the Rio Grande do Sul; shortly after, EI cases were detected in Porto Alegre, São Paulo, Pernambuco, Paraiba, and the Rio Grande do Norte, always after competition or exhibition affairs [25]. Thereafter, in September 2015, EIV was detected in vaccinated and unvaccinated horses suffering febrile respiratory disease at the Veterinary School Hospital, University of São Paulo, Brazil [43].

### 2.3. Uruguay

Although there is no information on clinical outcome or strength, and consequences of the disease to the equine industry, the available data reveals that the first event of EI in Uruguay occurred in September 1963 due to EIV of H3N8 subtype (A/eq/Uruguay/1/1963) [26,44,58]. Another occurrence of EI, for which only limited information could be obtained, occurred in 1976, but this time the infection was by EIV H7N7 subtype (A/eq/Uruguay/1063/1976). Later, in 2012, horses from all around the country were involved in multiple outbreaks of EI [24], during which almost the entire horse population at the Maroñas thoroughbred racing and training facilities (Montevideo) suffered a febrile acute respiratory disease, being more severe in young horses, and two deaths occurred [24]. Subsequently, in 2018, another outbreak of EI affected once again the entire equestrian activities in Uruguay; the EIV H3N8 subtype was identified in nasal swabs from diseased thoroughbred horses, both vaccinated and unvaccinated [26].

### 2.4. Argentina

Fain Binda et al. make a very detailed description of the first EI outbreak in Argentina, which occurred in 1976 due to the EIV H7N7 subtype [45]. Since then, all reported EI occurrences have been caused by the EIV H3N8 subtype. The first detection of the EIV H3N8 subtype in Argentina occurred in the summer of 1985–1986 and was described as one of the major outbreaks of EI owing to the rapid spread of the virus across the country [48]. Acute febrile respiratory disease with high morbidity occurred in 1993 among Thoroughbred horses stabled at the Palermo and San Isidro racecourses [16,51]. Sporadic H3N8 EIV incursions, with only a few horses involved, took place between 1994 and 2001 [17]. In 2005, EI was again described among horses housed in a jumping club in Buenos Aires city, which had temporarily received horses from Chile; morbidity was very low, approximately 10%, and, thanks to the early diagnosis and the immediate implementation of biosecurity measures, no cases outside this facility were reported [52].

Between July and November 2012, an extensive EI outbreak occurred in Argentina, starting two months after the EI outbreak in Uruguay. The movement of horses for racing purposes between Uruguay and Argentina is very common and is likely to have been the source of virus in Argentinian racetracks. Affected animals included thoroughbred, jumping and show horses and were identified all around the country in equestrian facilities located in 5 different provinces, 1000 km away from the index cases. EI vaccination before the movement of horses is mandatory in Argentina: hence, this EI outbreak took place in a regularly vaccinated horse population [24].

More recently, a huge outbreak of EI occurred in Argentina between March and July 2018, while EIV was affecting other countries in South America [23]. Again, during this EI occurrence, thoroughbred, polo and jumping horses in six different provinces around the country were involved. The index cases occurred in Mendoza province, which is geographically located in the border with Chile, suggesting the virus entered from Chile and it subsequently disseminated into the country. During this outbreak, the movement of horses, competitions and equestrian events were not officially restricted, favoring the spread of the virus throughout the country [23].

### 2.5. Colombia

Only scarce information is available on the occurrence of EI in Colombia. According to anecdotal evidence, in July 2005, an EIV outbreak occurred, leading to an interruption of horse movement (https://www.eltiempo.com/archivo/documento/MAM-1956702, accessed on 15 February 2021). Another EI event was reported in 2010 (https://www.ica.gov.co/getattachment/ICAComunica/PYP/influenzaequina/todo_sobre_influenza_equina.pdf.aspx?lang=es-CO, accessed on 15 February 2021). In 2018, EIV infection was confirmed by the Animal Health Authorities of Colombia (ICA) and reported to the OIE (https://oiebulletin.com/?officiel=08-4-1-2019-2-panel-en, accessed on 15 February 2021). An EIV H3N8 subtype was responsible for this outbreak of respiratory disease, which affected horses from different geographical locations; the outbreak started during a period of equestrian sporting events, which facilitated its rapid and widespread across the country. In Colombia, equine influenza vaccination is required for the national horse movement, especially for the animals attending events; however, there is no official vaccination campaign against this disease, with, therefore, an uncertain vaccine coverage (http://flu.org.cn/en/news-20,020.html, accessed on 15 February 2021).

### 2.6. Ecuador

A widespread outbreak of EI occurred in Ecuador in October 2018 (https://oiebulletin.com/?officiel=08-4-1-2019-2-panel-en, accessed on 15 February 2021). This was the first time that the virus was detected in the country, and clinical cases were observed in 10 premises involving 111 horses from a total population of 432; the index case was observed after an equestrian event (https://www.prosaia.org/influenza-equina-en-ecuador/#:~:text=El%20origen%20de%20la%20infecci%C3%B3n,PCR%20en%20el%20laboratorio%20nacional, accessed on 22 February 2021). The strain responsible for the outbreak was an H3N8 subtype (personal communication).

## 3. Molecular Evolution and Phylogenetic Relationship of H3N8 EIV Detected in South America

It is hypothesized that H3N8 EIV originated from an avian source in South America, where the avian to horse spillover took place with the subsequent horse-to-horse transmission and spread worldwide [2,17,59]. The origin in South America is suggested since the strain was identified for the first time in Miami, Florida, USA, early in 1963, after the arrival, by air, of thoroughbred horses from Argentina [10,14]. There is no information regarding the circulation of the EIV H3N8 subtype in Argentina before 1985, though H3N8 EIV infection and disease in horses in Chile, Brazil and Uruguay were described during that year [53,55]. Additionally, phylogeographic analysis suggests South America as the starting point, inferred by the earliest divergence events observed from Uruguay and Brazil to the USA between 1963 and 1969 [44].

**Figure 2:**
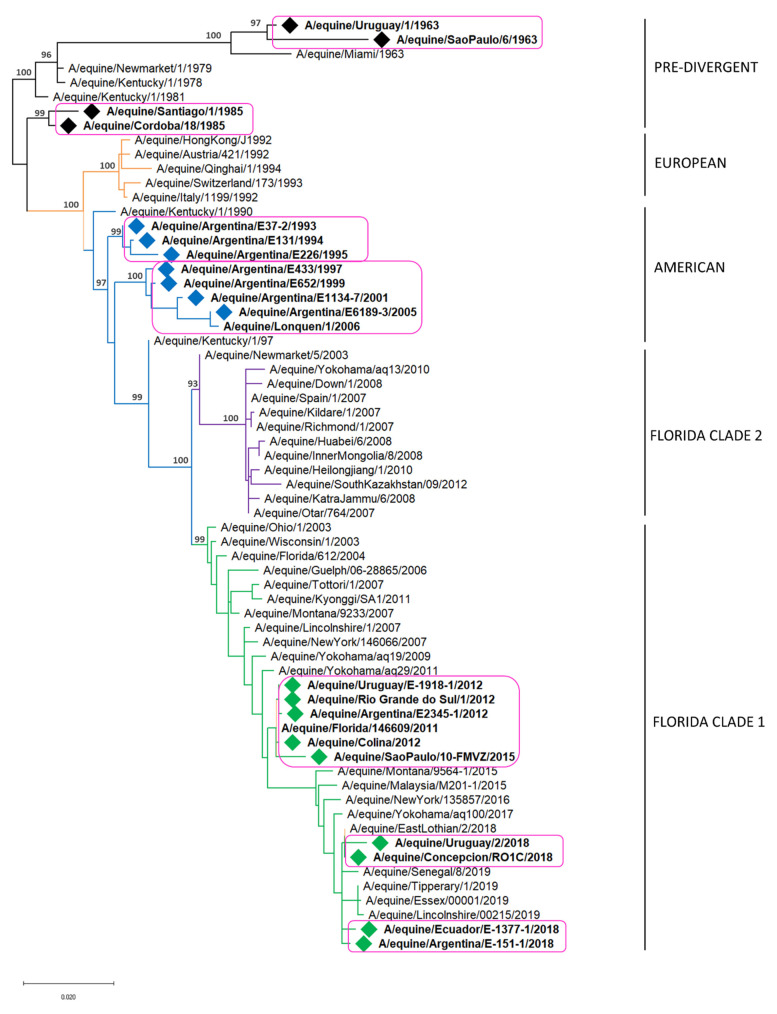
Maximum likelihood phylogenetic tree of the HA gene of H3N8 EIV. Bootstrap values obtained after 1000 replicates are shown at major nodes. Magenta boxes enclose South American strains. Colored diamonds (♦) represents the strains detected in South America: pre-divergent lineage (black), American lineage (blue), Florida clade 1 (green).

H3N8 EIV strains detected in South America, from the first detection in 1963 to the last in 2018, grouped into six monophyletic clades, all of them sustained by high support values (Figure 2) [44].

The strains detected until 1985 are included in the pre-divergent lineage, forming two different monophyletic clades, described as groups I and VIII [44]. Group I is made up of strains detected in Brazil in 1963 and 1969. Group VIII is made up of strains detected in Argentina, Chile and the USA in 1985. The USA strain included in this group could reflect the spread of the virus among subclinically infected horses traveling from South America to North America [44].

The strains detected from 1993–2006 grouped in the American lineage within the South American sublineage, which is also composed of two different monophyletic clades [17,44,52]. The South American clade 1 is composed only of Argentinian strains that circulated between 1993 and 1996 and have not been detected since then [44]. The South American clade 2 includes strains detected in Argentina and Chile between 1997 and 2006 [52]. The close resemblance of the viruses circulating in Argentina in 2005 and Chile in 2006 suggests that the virus entered Chile from Argentina [44,53].

During 2012, an extensive outbreak of EI occurred in different South American countries. The causative virus belonged to the Florida clade 1, and it was the first time this clade was detected in the region [24,25]. The first cases occurred in Chile at the beginning of the year; the virus then spread to Brazil [25] and later to Uruguay and Argentina [24,25]. All EIV detected in South America during the 2012 EI outbreak form a monophyletic cluster and are closely related with viruses isolated in the USA in 2011 (A/eq/Florida146609/11 strain), being then the USA strains the probable ancestor of the ones that circulated in South America in 2012. It is hypothesized that the spread was through direct horse movement from the USA to Chile rather than gradual spread from the Northern to the Southern countries [18,24,25,44]. The same virus was also detected in Dubai in 2012, in an outbreak of the disease in a quarantine facility after the entry of a group of endurance horses from Uruguay [18].

The strains circulating in Brazil in 2015 grouped together within the Florida clade 1 and showed similarities with the ones detected in other South American countries in 2012. According to evolutionary analyses, the 2012 and 2015 South American strains have the same ancestor. During the 2015 outbreak in Brazil, three genetic variants were identified (V1, V2 and V3), showing single amino acid substitutions at positions 121 and 304 of the HA1 (T-K, T-E and S-K, respectively), suggesting EIV evolved independently during the outbreak [43].

The causative virus of the multiple large-scale outbreaks in South America in 2018 also belongs to Florida clade 1. Nevertheless, the phylogenetic analysis allowed us to infer that the virus corresponds to a new introduction, probably into Chile, and it could be related to the ones circulating in Europe, Asia and North America in the same year [23,54]. The strains detected during the outbreak in Chile showed close similarities with EIV circulating in the UK in 2018 and Japan in 2017 [54]. Phylogenetic analysis carried out with the HA and NA genes of the strains detected later in Argentina showed that Argentinian strains grouped into a monophyletic group together with the isolates from Chile and share 100% amino acid sequence identity, suggesting that the source of the virus in Argentina could have been subclinically infected horses introduced from Chile [23]. Horses in Uruguay were also affected during this outbreak, and the molecular characterization of the partial HA gene demonstrated that the isolated strains displayed 99.6% nucleotide identity with the viruses detected previously in the same year in Argentina [26]. The HA gene characterization of the strains detected in Ecuador later this year showed 100% amino acid sequence identity with the ones detected previously in Chile, Argentina and Uruguay (personal communication). This information suggests that during the 2018 outbreak, the EIV spread from Chile to Argentina, and thereafter to Uruguay, and from these countries to other countries in South America.

As previously described, EI outbreaks that occurred in South America, from 1963 to the present, seem to be due to introducing new viruses into susceptible horse populations, probably through the movement of subclinically infected horses, especially at the international level, furthermore, facilitated by the short quarantine periods for horses traveling for competitions or sales [5,44,60]. The movement of horses for competition, reproductive and recreational purposes between Chile, Argentina, Uruguay and Brazil, is frequent, accelerating the spread of the virus in the region.

## 4. Equine Influenza Vaccination Status in South American Countries

Vaccination plays a major role in controlling EI infection and disease, and particularly for horses that travel frequently, intermingle with other horses and participate in shows, sport and competition events [5,6,37,61]. Almost in all South American countries, EI vaccination is mandatory for mobile or congregate equine populations [54,62].

Commercial vaccines for EI have been available for several decades worldwide. In South American countries, commercial vaccines are mostly imported vaccines produced by international pharmaceuticals, mainly Fluvac and Fluvac Innovator (Zoetis), and Prequenza TE (MSD). Some countries have local vaccine manufacturers, such as Biochemiq in Argentina (http://www.biochemiq.com/Producto/INFLUQIM, accessed on 25 March 2021), Veterquimica in Chile (https://www.veterquimica.cl/productos/cabolan/, accessed on 25 March 2021), Dechra in Brazil (https://www.dechra.com.br/detalhe-do-produto/83/influenza-horse, accessed on 25 March 2021), and so on. All these vaccines contain the whole inactivated EI virus. Thus, booster vaccination is required. Additionally, many of them contain out-of-date strains, which are not in line with the OIE-ESP recommendations; or have little information regarding immunogenicity and potency [23,54]. To illustrate this, we gathered information on vaccine coverage and vaccine performance in some of the EI outbreaks in South America previously described.

During the 2012 outbreak in Brazil, some animals had been vaccinated with products that contained out-of-date strains of EIV; it is, therefore, not surprising that these animals showed EI characteristic clinical signs. However, animals vaccinated with a whole inactivated product containing Florida clade 1 strain (as recommended by the ESP-OIE) were also affected [25]. Concerning the outbreak of 2015, 47% of the affected horses had been vaccinated. Among them, 67% had been vaccinated with a vaccine containing the out-of-date A/eq/Kentucky/1997 EIV strain [44].

During the last multifocal outbreak in Argentina in 2018, regularly vaccinated horses were affected, evidencing vaccine breakdown. Serological testing carried out in infected animals showed that 57% of them had moderate to high hemagglutination inhibition antibody titers (≥32) during the acute phase of the disease [23]. Additionally, 61% of the affected horses had up-to-date vaccination records according to National Animal Health Authorities regulation, yet 76% of them had been vaccinated with a vaccine containing the A/eq/Kentucky/1997 strain, which is phylogenetically and antigenically distant from the Florida clade 1 strain in circulation at that moment [23,60].

## 5. Conclusions-Perspectives

In the last 10 years, two main outbreaks, one in 2012 and the other in 2018, of EI occurred in Chile, Brazil, Colombia, Ecuador, Argentina and Uruguay, affecting both vaccinated and unvaccinated horses. There is no information on EI in other South American countries; this is impressive considering that, in general, outbreaks of EI in the region are characterized by being multifocal, starting in one country and subsequently spreading to others. This is likely to be due to a sub-notification of the disease in those countries or a lack of EIV diagnosis rather than to the absence of disease and viral circulation. This situation highlights the importance of not only carrying out epidemiological surveillance, diagnosis and characterization of the circulating EIV strains but also of homologating immunization requirements for international horse movement and harmonizing vaccine quality standards.

Given the importance of international transport of horses in the spread of EIV, and considering the significant economic impact that EI has on the equine industry, Health Authorities should promote incorporating a mandatory vaccination program for highly mobile horses, and at the same time, encourage horse vaccine producer companies to incorporate updated strains of EIV in line with OIE-ESP recommendations.

The described EI situation in South American countries emphasizes the need to implement appropriate quarantine and biosecurity measures since these are critical in preventing outbreaks of disease.

## Figures and Tables

**Table 1:** Summary of EIV outbreaks occurred in South America in chronological order.

Year of the Outbreak	Country	Subtype—Lineage	References
1963	Chile	H3N8—pre-divergent (group I)	[42]
	Brazil	H3N8—pre-divergent (group I)	[43]
	Uruguay	H3N8—pre-divergent	[26,44]
1969	Brazil	H3N8—pre-divergent (group I)	[25,43]
1976	Argentina	H7N7	[45]
1977	Chile	H7N7	[46]
	Brazil	H7N7	[43]
	Uruguay	H7N7	GenBank
1985	Chile	H3N8—pre—divergent (group VIII)	[47]
	Argentina	H3N8—pre—divergent (group VIII)	[48]
1986	Brazil	H3N8—n.d.	[49]
1988	Brazil	H3N8—n.d.	[49]
1992	Chile	H3N8—n.d.	[47,50]
1993	Argentina	H3N8—South American clade 1	[16,51]
1994	Argentina	H3N8—South American clade 1	[17]
2001	Argentina	H3N8—South American clade 2	[17]
2005	Argentina	H3N8—South American clade 2	[52]
2006	Chile	H3N8—South American clade 2	[53]
2008	Brazil	H3N8—n.d.	[43]
2010	Brazil	H3N8—n.d.	[43]
2012	Chile	H3N8—Florida clade 1	OIE
	Brazil	H3N8—Florida clade 1	[25]
	Uruguay	H3N8—Florida clade 1	[24]
	Argentina	H3N8—Florida clade 1	[24]
2015	Brazil	H3N8—Florida clade 1	[43]
2018	Chile	H3N8—Florida clade 1	[54]
	Argentina	H3N8—Florida clade 1	[23]
	Uruguay	H3N8—Florida clade 1	[26]
	Ecuador	H3N8—Florida clade 1	This report

n.d.: information regarding lineage was not determined.

## Data Availability

Data sharing not applicable.
